# Sleep Duration and Bedtime in the PURE Poland Cohort Study and the Link with Noncommunicable Diseases

**DOI:** 10.3390/ijerph19010403

**Published:** 2021-12-30

**Authors:** Katarzyna Zatońska, Alicja Basiak-Rasała, Katarzyna Połtyn-Zaradna, Krystian Kinastowski, Andrzej Szuba

**Affiliations:** 1 Department of Population Health, Wroclaw Medical University, 50-345 Wroclaw, Poland; katarzyna.zatonska@umw.edu.pl (K.Z.); katarzyna.poltyn-zaradna@umw.edu.pl (K.P.-Z.); 2 Calisia University, 62-800 Kalisz, Poland; krystian@kinastowski.pl; 3 Department of Angiology, Hypertension and Diabetology, Wroclaw Medical University, 50-529 Wroclaw, Poland; andrzej.szuba@umw.edu.pl

**Keywords:** sleep duration, bedtime, noncommunicable diseases, cohort study

## Abstract

(1) Background: The objective was to investigate the association between sleep duration, bedtime, and noncommunicable diseases in the PURE Poland cohort study. (2) Methods: The baseline study was conducted in 2007–2010. The study group comprised 2023 adult inhabitants of urban and rural areas in Lower Silesia, Poland. The study protocol included questionnaires, blood pressure measurements, blood draws, and anthropometric measurements. Sleep duration and bedtime were self-reported. (3) Results: The median sleep duration of women was 30 min longer than men (8 h vs. 7.5 h; *p* = 0.001). The average time of sleep increased along with the age of the participants. A sleep duration of >8 h was more common in rural than in urban participants (40.2% vs. 27.1%; respectively; *p* < 0.001). The relative risk of diabetes, stroke, hypertension, cardiovascular diseases (CVD), and obesity was significantly higher in participants who went to bed between 6 p.m. and 10 p.m. in comparison to those who went to bed between 10 p.m. and 12 a.m. (RR 2.23, 95% CI 1.06–4.67; RR 2.52, 95% CI 1.28 to 4.97; RR 1.12, 95% CI 1.04–1.20; RR 1.36; 95% CI 1.1–1.68; RR 1.38; 95% CI 1.15–1.66, respectively). The relative risk of respiratory diseases was two-fold higher in those who went to bed after midnight in comparison to those who went to bed between 10 p.m. and 12 a.m. (RR 2.24; 95% CI 1.19–4.22). (4) Conclusions: In our study, an earlier bedtime was associated with a higher risk of diabetes, stroke, obesity, hypertension, and CVD.

## 1. Introduction

Sufficient sleep is one of the key contributors to proper functioning and wellbeing. Sleep deprivation affects our immune system, metabolism, and mental health [1]. According to the American Academy of Sleep Medicine (AASM) and the Sleep Research Society (SRS), adults should sleep approximately 7 h per night, since a shorter duration of sleep can be associated with adverse health outcomes [2]. The AASM and the SRS guidelines recognize also that sleep duration exceeding 9 h per night may be beneficial for young adults, but it is uncertain if a longer duration of sleep is associated with health outcomes in adults [2]. On the other hand, both short and long sleep durations have been associated with an increased risk of mortality, type 2 diabetes, cardiovascular diseases (CVD), stroke, coronary heart disease (CHD), and obesity [3,4,5,6,7,8]. Short sleep duration in middle age has been also associated with an increased risk of dementia [9]. A sleep duration of 6–8 h per day was considered optimal and was associated with the lowest risk of mortality and CVD events [4]. The risk of short sleep duration and sleep disturbances increases with increasing age [10]. Older age has been also associated with earlier bedtime and earlier wake up [11]. Not merely the sleep duration but also the bedtime has been associated with an increased risk of metabolic disorders. Knutson et al. [12] reported an association between later sleep timing and a higher risk of insulin resistance, especially in older participants. The association between sleep duration or bedtime and CVD events and mortality has been recently investigated in the global Prospective Urban and Rural Epidemiology Study (PURE) [4,8].

According to the results of the NHANES study, the average US citizen sleeps approximately 6.9 h per night [13]. The European average sleep duration equals 7.1 h per night [14]. In general, women tend to sleep longer than men [15,16]. According to the report of the Public Opinion Research Center (CBOS), 49% of Poles sleep fewer than 6 h per night at least once a week [17]. A total of 8% of Poles regularly sleep fewer than 6 h per night [17]. Sleeping patterns and the association between bedtime and noncommunicable diseases (NCDs) are rarely researched in Poland. The literature published so far has focused mainly on sleep disturbances, obstructive sleep apnea, and bruxism. One of the largest studies conducted by Kiejna et al. [18,19] presented the results of the National Health Interview Survey. The study focused mainly on sleep disturbances, including insomnia. According to the study, one-fourth of the population suffered from insomnia, and the risk of sleep disturbances increased with age [19]. The mean duration of sleep was 7.7 h with no difference between women and men [18]. Self-reported sleep problems were also investigated in a study focusing on the risk factors for cardiovascular diseases, NATPOL [20]. Sleep duration, sleep disturbances, and bedtime were investigated in Poland in a sample of pregnant women [21]. Overweight and obesity have been associated with sleep disturbances in a study by Lizończyk et al. [22] conducted in a group of adolescents.

Poland is one of the 21 countries enrolled in the PURE study. To our knowledge, our study is one of the first of this scale in Poland investigating the relationship between the duration of sleep, bedtime, and the risk of NCDs in adults.

## 2. Materials and Methods

The baseline data were collected between 2007 and 2010. A Polish cohort was enrolled into the global Prospective Urban and Rural Epidemiological Study (PURE). All participants were examined in accordance with the global PURE study protocol [23], which included a questionnaire study (individual health, household, family, food frequency questionnaire, and international physical activity questionnaire (IPAQ)), anthropometric measurements, blood pressure measurement, blood draw, ECG, and spirometry. The study design and characteristics of the baseline Polish cohort were described in detail elsewhere [24]. The baseline cohort consisted of 2035 adult participants (1281 women and 754 men) aged 30–85 years (mean: 55 years, SD ± 10). The study group comprised participants from urban (the city of Wrocław) and rural (villages surrounding Wrocław) areas in Lower Silesia, Poland.

Sleep duration and bedtime were self-reported by the participants. There were three questions in the individual health questionnaire, which directly referred to sleeping habits [23]:

(1) During your longest or nocturnal sleep period, what time do you normally go to bed?
(2) During your longest or nocturnal sleep period, what time do you normally wake up?
(3) Do you usually take naps/siestas?

In the case of (1) and (2), the participants were asked to indicate the approximate hour of bedtime and wake up (00:00–23:59). If participants reported taking naps in (3), they were asked to assess the nap duration (in minutes). Total sleep duration per day was defined as the sum of estimated nocturnal sleep time and self-reported nap duration. Considering the total duration of sleep, we divided the participants into three groups: (1) <6 h of sleep, (2) 6–8 h of sleep, and (3) >8 h of sleep. Following the approach presented by Wang et al. [8], introducing 10:00 p.m. and 00:00 a.m. (midnight) as cut-offs, the participants were divided into three groups: (1) bedtime before 10 p.m., (2) bedtime between 10 p.m. and midnight, and (3) bedtime after midnight.

The body mass of the participants was measured with the use of a Tanita Ironman Body Composition Monitor Model BC-554 with an accuracy of 0.1 kg. The Body Mass Index (BMI) was calculated as weight (kg) divided by height (m) squared. Participants were divided into four BMI categories, according to the WHO guidelines: underweight (BMI < 18.5 kg/m^2^), normal weight (BMI 18.5–24.9 kg/m^2^), overweight (BMI 25.0–29.9 kg/m^2^), and obese (BMI ≥ 30.0 kg/m^2^). Diabetes was ascertained on the basis of self-reported diabetes and/or self-reported anti-diabetic medication and/or fasting blood glucose measurement ≥ 126 mg/dL [25]. Hypertension was ascertained on the basis of self-reported hypertension and/or self-reported anti-hypertensive medication and/or an average of two blood pressure measurements ≥ 140/90 mmHg as previously described [26]. The occurrence of cardiovascular diseases (CVD) and respiratory diseases was self-reported by the participants. The category of CVD comprised participants who reported heart failure, coronary heart disease, and other heart diseases. Respiratory diseases comprised asthma and chronic obstructive pulmonary disease (COPD). Attitudes toward tobacco smoking and alcohol consumption were self-reported by the participants. In the case of tobacco smoking, participants could have chosen one of three possible answers: “formerly used tobacco products”, “currently use tobacco products”, or “never used tobacco products”. Similarly, in the case of the question regarding alcohol consumption (“Which best describes your history of alcohol use?”), participants could have chosen between “formerly used alcohol products”, “currently use alcohol products”, and “never used alcohol products”. The study has been reviewed and accepted by the Bioethics Committee of the Wrocław Medical University and have therefore been performed in accordance with the ethical standards laid down in an appropriate version of the 1964 Declaration of Helsinki (positive opinion of The Bioethics Committee of the Wrocław Medical University nr KB-443/2006).

Sleeping patterns in the PURE Poland cohort study were described using basic statistical parameters. The Kolmogorov–Smirnov test with the Lillefors correction and the Shapiro–Wilk test were used to assess the normality of the distributions of the analyzed variables. As empirical distributions significantly deviated from the normal distribution, non-parametric tests were used to assess the significance of differences in sleep duration between the studied groups: for two groups, the Mann–Whitney U test was used, and when there were more groups, the Kruskal–Wallis test was used. The Pearson Chi-square test was used to assess the independence between the two variables. The relative risk (RR) with 95% confidence intervals (CI) was calculated to take into account the risk of the occurrence of the selected NCD in groups of participants differentiated according to bedtime. The relative risk was calculated directly from the contingency tables (C × R). A bedtime between 10 p.m. and 12 a.m. was considered a reference value. In all analyses, the level of statistical significance was set at *p* ≤ 0.05. Statistical analysis was performed using the Statistica 13.3 software (TIBCO. Software Inc., Palo Alto, CA, USA).

## 3. Results

A total of 2023 participants were enrolled in the analysis. Eleven participants were excluded due to a lack of information on bedtime. Shift workers who declared bedtime between 4 a.m. and 12 p.m. (5 participants) were also excluded.

### 3.1. Total Sleep Duration

The characteristics of the total sleep duration in our study are presented in Table 1. There was a significant difference in total sleep duration between men and women (*p* < 0.001). Women slept an average of 7.9 h ± 1.4, whereas men 7.7 h ± 1.3. The median sleep duration of women was approximately 30 min longer than men (8 h vs. 7.5 h; *p* = 0.001). Among women, 9.1% reported sleep duration < 6 h, 55.5% reported 6–8 h, and 35.4% reported > 8 h of sleep. Among men, 11.9% reported sleep duration < 6 h, 60.7% reported 6–8 h of sleep, and 27.4% reported > 8 h of sleep. Total sleep duration was significantly differentiated by sex, age, place of residence, education, professional activity, bedtime, attitudes toward tobacco smoking and alcohol drinking, level of physical activity, WHR, and the occurrence of chosen non-communicable diseases. In our study, the total sleep duration was longer in older participants. The average time of sleep in 30- to 44-year-olds was significantly shorter than in 45- to 64-year-olds (7.5 h vs. 7.8 h, respectively; *p* = 0.024) and shorter than in participants > 64 years of age (7.5 h vs. 8.5 h; *p* < 0.001). Moreover, 45- to 64-year-olds also slept shorter than participants > 64 years of age (7.8 h vs. 8.5 h, respectively; *p* < 0.001). Every 10 years, the duration of sleep increased, on average, by 0.3 h. An earlier bedtime was associated with a longer sleep duration. Participants who declared a bedtime between 6 p.m. and 10 p.m. slept, on average, 8.6 h ± 1.1; participants who declared bedtime between 10 p.m. and 0 a.m. slept, on average, 7.4 h ± 1.1; and those who went to bed between midnight and 4 a.m. slept, on average, 6.5 h ± 1.4; *p* < 0.001.

Sleep duration has been differentiated by the place of residence and level of education (*p* < 0.001). Among urban participants, 12.1% slept <6 h, 60.8% slept 6–8 h, and 27.1% slept more than 8 h. In comparison, among rural participants, 7.3% slept < 6 h, 52.5% slept 6–8 h, and 40.2% slept more than 8 h. Considering the level of education, 4.2% of participants with primary or unknown education slept < 6 h, 41.2% slept 6–8 h, and 54.6% slept more than 8 h, respectively. The 11.6% of participants with higher education reported fewer than 6 h of sleep, 66.4% reported 6–8 h of sleep, and 22.0% reported more than 8 h of sleep.

Total sleep duration was not significantly differentiated by BMI (*p* = 0.059). On the other hand, an average BMI was slightly higher among participants who slept, on average, > 8 h per night, followed by those who slept 6–8 h (mean 28.5 kg/m^2^ ± 5.2 and 28.0 kg/m^2^ ± 5.0, respectively). The mean WHR was the highest among those who slept more than 8 h (0.90 ± 0.1; *p* = 0.005). The mean systolic blood pressure (SBP) was the highest in participants who slept more than 8 h (147 ± 23; *p* = 0.015). On the other hand, heart rate was higher in those who slept < 6 h (74 ± 13; *p* = 0.042).

### 3.2. Naps

Taking naps during the day was also more prevalent in men than women (38.7% vs. 27.1%, respectively; *p* < 0.001). The duration of the naps of those participants who slept 6–8 h was, on average, 9 min shorter than of those who slept < 6 h (38.5 min vs. 47.5 min; *p* = 0.015). Patients who took naps were older by 2 years than those who did not take naps (56.1 min ± 9.7 vs. 53.9 min ± 9.8; *p* = 0.001). Naps were significantly more common in older participants.

### 3.3. Total Sleep Duration and Bedtime According to Chosen Non-Communicable Diseases

**Figure 1:**
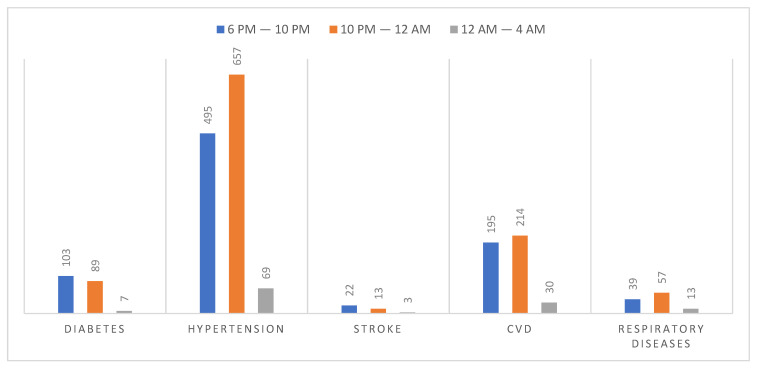
Bedtime in participants with chosen noncommunicable diseases. In the case of every disease, there were statistically significant differences between the categories differentiated by bedtime (*p* < 0.02). CVD—cardiovascular diseases.

A total of 13% of participants went to sleep between 6 p.m. and 10 p.m., 71% went to sleep between 10 p.m. and 12 a.m., and 17% between 12 a.m. and 4 a.m. The basic characteristics of bedtime according to chosen non-communicable diseases are presented in Figure 1. A bedtime between 6 p.m. and 10 p.m. was more common for participants with diabetes and stroke, whereas a bedtime between 10 p.m. and 12 a.m. was more prevalent in participants with hypertension, CVD, and respiratory diseases.

Among participants with diabetes, 50.8% slept > 8 h, 43.2% slept 6–8 h and 6.0% slept fewer than 6 h, respectively (*p* < 0.001). The relative risk of diabetes was two-fold higher and of stroke 2.5-fold higher in participants who went to bed between 6 p.m. and 10 p.m., in comparison to those who went to bed between 10 p.m. and 12 a.m. [RR 2.23; 95% CI 1.06–4.67; RR 2.52; 95% CI 1.28 to 4.97].

Among participants with diabetes, 50.8% slept > 8 h, 43.2% slept 6–8 h, and 6.0% slept fewer than 6 h, respectively (*p* < 0.001). The relative risk of diabetes was two-fold higher and of stroke 2.5-fold higher in participants who went to bed between 6 p.m. and 10 p.m., in comparison to those who went to bed between 10 p.m. and 12 a.m. (RR 2.23; 95% CI 1.06–4.67; RR 2.52; 95% CI 1.28 to 4.97).

Among participants with hypertension, 35.3% slept > 8 h, 54.5% slept 6–8 h, and 10.3% slept fewer than 6 h, respectively (*p* = 0.002). The relative risk of hypertension was significantly higher in participants who went to bed between 6 p.m. and 10 p.m., in comparison to those who went to bed between 10 p.m. and 12 a.m. (RR 1.12; 95% CI 1.04–1.20).

Among participants with CVD, 42.4% slept > 8 h, 48.3% slept 6–8 h, and 9.3% slept fewer than 6 h, respectively (*p* < 0.001). Moreover, the relative risk of CVD was also significantly higher in those participants who went to bed between 6 p.m. and 10 p.m., in comparison to those who went to bed between 10 p.m. and midnight (RR 1.36; 95% CI 1.1–1.68).

The relative risk of obesity according to BMI was significantly higher in participants, who went to bed between 6 p.m. and 10 p.m., in comparison to those who went to bed between 10 p.m. and 12 a.m. (RR 1.38; 95% CI 1.15–1.66).

**Figure 2:**
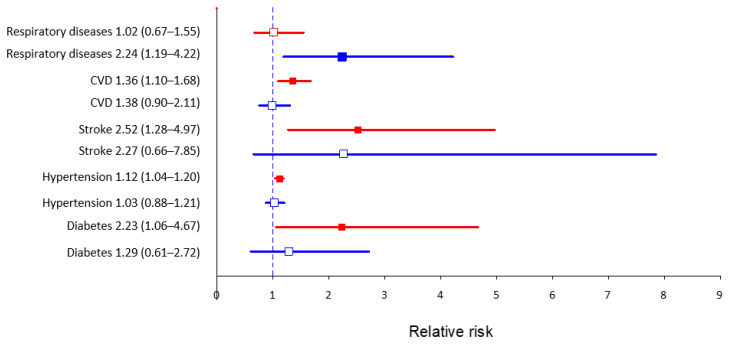
Relative risk with 95% confidence intervals of the occurrence of chosen non-communicable diseases in participants who went to sleep earlier (6 p.m.–10 p.m., indicated by the red color) and later (12 a.m.–4 a.m., indicated by the blue color) in comparison to those who went to sleep between 10 p.m. and 12 a.m. (reference value).

On the other hand, the relative risk of respiratory diseases was two-fold higher in those who went to bed after midnight, in comparison to those who went to bed between 10 p.m. and 12 a.m. (RR 2.24; 95% CI 1.19–4.22). Participants with respiratory diseases were not significantly differentiated by sleep duration. The analysis of relative risk with 95% confidence intervals of the occurrence of chosen non-communicable diseases in participants who went to sleep earlier (6 p.m.–10 p.m.) and later (12 a.m.–4 a.m.) in comparison to those who went to sleep between 10 p.m. and 12 a.m. is presented in Figure 2.

## 4. Discussion

Deprivation and low quality of sleep have been linked to many metabolic diseases, including obesity and type 2 diabetes. The presented article aimed to characterize sleep duration and bedtime in the PURE Poland cohort study in the view of sociodemographic characteristics and chosen NCDs. The PURE Poland study is one of the few longitudinal cohort studies in Poland. In our cohort, sleep duration was significantly differentiated by sociodemographic factors (sex, age, place of residence, level of education, professional activity status), behavioral factors (bedtime, taking naps, tobacco smoking, alcohol drinking), anthropometric factors (BMI, WHR), and some NCDs (diabetes, hypertension, stroke, CVD). The relative risk of diabetes, obesity, hypertension, stroke, and CVDs was significantly higher in participants who went to bed between 6 p.m. and 10 p.m. in comparison to the control (bedtime 10 p.m.–12 a.m.).

In our cohort, the average duration of sleep was significantly longer in women than in men. On the other hand, more men than women declared taking naps during the day. Conforming to our results, in the global PURE study analysis, a longer duration of sleep was more prevalent in women, participants > 50 years old, and in those living in rural areas [8]. In our cohort, there were more long sleepers among the participants with primary education.

In our study, a shorter sleep duration was more common in current smokers and alcohol drinkers. Similarly, in the Whitehall study [27], a shorter sleep duration was more common in participants consuming alcohol in comparison to never-drinkers.

Too-short and too-long durations of sleep have been previously associated with an increased risk of type 2 diabetes [28,29]. In a study published by Cappuccio et al. [28], a long duration of sleep (>8–9 h/night) was associated with a relative risk (RR) of type 2 diabetes of 1.48 [CI 1.13–1.96]. Both short and long durations of sleep have been associated with increased risk of CHD, stroke, and total CVD [30]. In a prospective study and meta-analysis by Leng et al. [31], a long duration of sleep has been significantly associated with an increased risk of stroke. On the other hand, in a meta-analysis of prospective cohort studies conducted by Li et al. [32], both short and long durations of sleep were associated with an increased risk of stroke, but a long sleep duration was a significant marker of stroke mortality. In our cohort, hypertensive participants had a tendency for a longer duration of sleep, which is consistent with the results from the global PURE study [8]. It is speculated that fatigue or a longer duration of sleep can be a preliminary symptom of slowly developing health problems, hence the significant association between a long duration of sleep and mortality or morbidity observed in the studies [8]. Having said that, we cannot determine the causality at this stage. In our cohort, the duration of sleep increased along with the increasing age of participants. As a general principle, aging is associated with disturbed sleep, a decreased ability to maintain sleep, and a shorter sleep duration [33]. In our study, total sleep duration combined both nocturnal sleep and the sum of daytime naps, which may have contributed to this association. It has been previously observed that the frequency of daytime napping increases with age [34]. There were also studies that indicated that retirement promoted longer sleep duration [35]. In a study by Basner et al. [36], participants who were unemployed or retired reported longer sleep duration and were less likely to be short sleepers.

In our study, an earlier bedtime was associated with a higher risk of the chosen NCDs, including diabetes and hypertension. On the contrary to our results, in a study by Yan et al. [37], a later bedtime (after midnight) was associated with an increased prevalence of diabetes (OR 1.446; 95% CI 1.107–1.888]. A U-shaped relationship between the prevalence of hypertension and bedtime was observed by Jansen et al. [38]. The risk of hypertension was almost two-fold higher in those participants who went to bed before 9 p.m., as well as after 11 p.m., in comparison to those, who went to bed between 9 p.m. and 11 p.m. (RR 1.96; 95% CI 1.27–3.01; RR1.87; 95% CI 1.09–2.21, respectively) [38]. Similarly, a U-shaped relationship between bedtime and health outcomes was observed in the global PURE study [8]. Both those who went to bed early (before 10 p.m.) and late (after midnight) had a higher risk of mortality and major CVD events [8]. In our study, the relative risk of respiratory diseases was two-fold higher in those who went to bed after midnight in comparison to those who went to bed between 10 p.m. and 12 a.m. The direct association between bedtime and respiratory diseases has been rarely described in the literature. However, the diurnal variability of lung function can influence bedtime, the duration of sleep, and the quality of sleep in asthmatic patients [39,40]. A nocturnal decline in pulmonary function has been previously associated with later bedtime and shorter sleep duration [41]. Chronic obstructive pulmonary disease (COPD) has been also associated with disturbed sleep, insomnia, and delayed initiation of sleep [42], which may partially explain the association observed in our study.

There are some limitations of our study to discuss. The current analysis focuses solely on data collected at the baseline. Our study is a cohort study, so the results should be treated with caution. Our cohort is characterized by the overrepresentation of women and elderly participants in comparison to the general Polish population. A lot of participants have already retired, which influences their sleeping patterns. Sleep disorders, including insomnia, were not investigated in the PURE study. The use of medication, which may influence sleeping patterns, was not investigated in this analysis. On the other hand, there are also several strengths to discuss. Our study is one of the few cohort studies in Poland of this size. To our knowledge, our study is one of the first of this scale in Poland investigating the relationship between the duration of sleep, bedtime, and the risk of NCDs in adults. It is planned that future research will include a longitudinal analysis of bedtime and duration of sleep in the study population.

## 5. Conclusions

Sleep duration and bedtime were significantly differentiated by sociodemographic and behavioral factors. In our study, an earlier bedtime was associated with a higher risk of diabetes, obesity, hypertension, and CVD.

## Figures and Tables

**Table 1:** Total sleep duration in the study population.

Variable	Total Sleep Duration (Hours)			
	<6 *n* = 205	6–8 *n* = 1162	>8 *n* = 656	*p*-Value **
Age (years), Me (Q1, Q3)	53 (45, 57)	54 (47, 60)	60 (52, 65)	<0.001
Sex				
Female, *n* (%)	116 (9.1)	707 (55.5)	451 (35.4)	<0.001
Male, *n* (%)	89 (11.9)	455 (60.7)	205 (27.4)	
Place of residence				
Urban, *n* (%)	145 (12.1)	730 (60.8)	325 (27.1)	<0.001
Rural, *n* (%)	60 (7.3)	432 (52.5)	331 (40.2)	
Marital status				
Never married, *n* (%)	18 (12.3)	92 (63.0)	36 (24.7)	0.057
Married/living together, *n* (%)	152 (10.1)	870 (58.0)	478 (31.9)	
Separated/divorced/widowed, *n* (%)	35 (9.3)	200 (53.1)	142 (37.6)	
Education				
Primary or unknown, *n* (%)	13 (4.2)	126 (41.2)	167 (54.6)	<0.001
Vocational, *n* (%)	25 (7.7)	193 (59.6)	106 (32.7)	
Secondary, *n* (%)	97 (12.2)	444 (56.1)	251 (31.7)	
Higher, *n* (%)	70 (11.6)	399 (66.4)	132 (22.0)	
Professional activity				
Professionally active, *n* (%)	161 (14.5)	756 (67.9)	196 (17.6)	<0.001
Retired, *n* (%)	35 (5.0)	316 (45.0)	352 (50.0)	
Pensioner, *n* (%)	9 (4.3)	90 (43.5)	108 (52.2)	
Bedtime				
6 p.m.–10 p.m., *n* (%)	7 (1.0)	348 (45.4)	411 (53.6)	<0.001
10 p.m.–0 a.m., *n* (%)	147 (12.9)	760 (66.6)	234 (20.5)	
0 a.m.–4 a.m., *n* (%)	51 (43.9)	54 (46.6)	11 (9.5)	
Naps				
Taking naps, *n* (%)	74 (11.7)	346 (54.6)	214 (33.7)	0.137
Average nap time (min), M ± SD	47.5 ± 36.4	38.5 ± 24.3	42.9 ± 27.0	0.015
Attitudes toward tobacco smoking				
Never smokers, *n* (%)	89 (9.3)	520 (54.3)	349 (36.4)	<0.001
Former smokers, *n* (%)	59 (9.2)	401 (62.5)	182 (28.3)	
Current smokers, *n* (%)	57 (13.5)	241 (57.0)	125 (29.6)	
Attitudes toward alcohol consumption				
Never drinkers, *n* (%)	44 (9.7)	240 (53.1)	168 (37.2)	<0.001
Former drinkers, *n* (%)	18 (8.7)	99 (47.6)	91 (43.6)	
Current drinkers, *n* (%)	143 (10.5)	823 (60.4)	397 (29.1)	
Physical activity, MET × min/week *	*N* = 190	*N* = 1063	*N* = 612	
Low (<600), *n* (%)	3 (4.9)	33 (54.1)	25 (41)	0.157
Moderate (600–3000), *n* (%)	48 (9.4)	281 (54.9)	183 (35.7)	
High (>3000), *n* (%)	139 (10.8)	749 (58.0)	404 (31.3)	
Blood pressure				
SBP (mm Hg), Mean ± SD	145 ± 19	144 ± 21	147 ± 23	0.015
DBP (mm Hg), Mean ± SD	87 ± 11	86 ± 12	86 ± 11	0.314
HR (bmp), Mean ± SD	74 ± 13	72 ± 10	72 ± 11	0.042
Body Mass Index (BMI)				
BMI (kg/m^2^), Mean ± SD	27.9 ± 4.8	28.0 ± 5.0	28.5 ± 5.2	0.059
Underweight, *n* (%)	1 (6.6)	7 (46.7)	7 (46.7)	0.271
Normal, *n* (%)	61 (10.7)	336 (58.9)	173 (30.4)	
Overweight, *n* (%)	84 (10.4)	476 (58.9)	248 (30.7)	
Obesity, *n* (%)	59 (9.4)	343 (54.4)	227 (36.2)	
Waist-to-hip ratio (WHR)				
WHR (-), Mean ± SD	0.884 ± 0.094	0.880 ± 0.094	0.900 ± 0.100	0.005
Normal, *n* (%)	78 (11.0)	449 (63.3)	182 (25.7)	<0.001
Central obesity, *n* (%)	127 (9.7)	713 (54.3)	474 (36.1)	
Noncommunicable diseases				
Diabetes, *n* (%)	12 (6.0)	86 (43.2)	101 (50.8)	<0.001
Hypertension, *n* (%)	125 (10.3)	664 (54.5)	430 (35.3)	0.002
Cardiovascular diseases, *n* (%)	41 (9.3)	212 (48.3)	186 (42.4)	<0.001
Respiratory diseases, *n* (%)	14 (12.8)	60 (55.1)	35 (32.1)	0.621

* Physical activity, unlike other variables, was assessed in the group of 1865 participants, the number of participants in categories differentiated by sleep duration in this analysis has been placed in the same verse; ** statistically significant *p*-value has been highlighted in bold. Abbreviations: Me—median, M—mean; SD—standard deviation; SBP—systolic blood pressure; DBP—diastolic blood pressure; HR—heart rate; BMI—Body mass index; WHR—waist to hip ratio.

## Data Availability

Data available on request due to restrictions, e.g., privacy or ethical.
